# Cellular events and behaviors after grafting of stratified squamous epithelial cell sheet onto a hydrated collagen gel

**DOI:** 10.1002/2211-5463.12213

**Published:** 2017-03-28

**Authors:** Yoshiyuki Kasai, Naoya Takeda, Shinichiro Kobayashi, Ryo Takagi, Masayuki Yamato

**Affiliations:** ^1^ Department of Life Science and Medical BioscienceGraduate School of Advanced Science and EngineeringWaseda University (TWIns)Shinjuku‐kuTokyoJapan; ^2^ Institute of Advanced Biomedical Engineering and ScienceTokyo Women's Medical University (TWIns)Shinjuku‐kuTokyoJapan; ^3^ Department of SurgeryNagasaki University Graduate School of Biomedical SciencesNagasaki‐shiNagasakiJapan

**Keywords:** collective cell migration, grafting, partial EMT, regenerative therapy, stratified squamous epithelial cell sheet

## Abstract

Autologous stratified squamous epithelial cell sheets have been successfully used to treat epithelial defects in tissues such as the cornea and the esophagus. However, the regenerative cellular events occurring in the grafted epithelial cells are unclear in the early stages of wound healing. In this study, we created an *in vitro* grafting model using cultured normal human epidermal keratinocyte (NHEK) sheets and a type I collagen gel to investigate the cellular processes that occur within the grafted cell sheet. Cultured NHEK cells successfully became a stratified squamous cell sheet resembling epithelial tissue, retained expression of cellular integrins and adhesion proteins, and adhered successfully to a type I collagen gel. After culture on the collagen gel, expression of E‐cadherin, and β‐catenin decreased in the cells of the basal layer of the grafted cell sheet, resembling events characteristic of a partial epithelial–mesenchymal transition (EMT). These basal cells also induced migration of the cell sheet. Those phenomena are consistent with the essential events that occur in the wound‐healing process observed previously in cell studies. Therefore, the epithelial cell sheet grafted onto a type I collagen gel is a suitable model *in vitro* to study cellular events and behaviors. Furthermore, we also addressed the therapeutic mechanisms by which the epithelial cell sheet promotes wound healing.

**Abbreviations**
EMT: epithelial–mesenchymal transition
ESD: endoscopic submucosal dissection
IHC: immunohistochemistry
KCM: keratinocyte culture medium
NHEK: normal human epidermal keratinocyte
PIV: particle image velocimetry
qRT‐PCR: quantitative real‐time PCR

Transplantable grafts of autologous stratified squamous epithelial cells have been successfully used to treat epithelial defects in the skin, cornea, and esophagus. In particular, we have fabricated transplantable epithelial cell sheets composed of a patient's own oral mucosal epithelial cells using temperature‐responsive culture surfaces, and these cell sheets have been used to treat patients suffering from total corneal epithelial stem cell deficiency 1, and to prevent stricture of the esophagus after endoscopic submucosal dissection (ESD) 2, 3. In order to make these functional culture surfaces, the temperature‐responsive polymer poly‐(*N*‐isopropylacrylamide) is covalently immobilized onto a plastic tissue culture plate. Cultured cells can then adhere, spread, and proliferate on these surfaces at 37 °C, and can be detached when the temperature is reduced to below 32 °C 4.

Intact cell sheets can be harvested with a simple temperature reduction without the need for proteolytic enzymes. The harvested cell sheets retain their cell–cell junction proteins, like cadherin, membrane proteins including growth factor receptors and ion channels, as well as the extracellular matrix (ECM) secreted during culture 5. This retained structure allows transplantable epithelial cell sheets to readily adhere to the wound beds of the esophageal stroma even in the presence of potentially disruptive tissue movements, like the peristaltic movement of the esophagus that accompanies the heartbeat. Grafted cell sheets integrate within a short period, removing the need for any glue, suturing, or clipping. Using this cell sheet therapy in a swine model, we have reported complete healing of an esophageal ulcer with re‐epithelialization within 2 weeks after grafting onto an ESD wound bed. Fluorescent labeling of grafted epithelial cell sheets revealed that the grafted epithelial cells remained at the grafted site at least 1 week after grafting 6. However, the precise cellular events and the dynamic changes that occur following grafting of the cell sheets onto the wound bed are still not understood. Therefore, there is a clear need to better understand how the cell sheet achieves its wound‐healing effect.

In this study, we established an *in vitro* grafting model to analyze behaviors of distinct cell layers in a stratified squamous epithelial cell sheet. Normal human epidermal keratinocyte (NHEK) cells were used in this study because they exhibit characteristics of both stratified squamous epithelial cell and oral mucosal epithelial cell 6, and are commercially available. We used a simple type I collagen gel matrix as the grafting site. We focused on the partial epithelial–mesenchymal transition (EMT) events in each layer, and examined the spatiotemporal expression of marker proteins that are indicative of epithelial‐specific cell–cell adhesion (e.g., E‐cadherin) as well as mesenchymal markers (e.g., vimentin).

## Materials and methods

### Construction of NHEK cell sheets

Keratinocyte culture medium (KCM) was prepared according to a method previously described 7, 8. Dulbecco's modified Eagle's medium‐high glucose (DMEM; Sigma‐Aldrich, MO, USA) and Ham's F‐12 (Sigma‐Aldrich) were mixed at a 3 : 1 (v/v) ratio. The medium was supplemented with 100 IU·mL^−1^ penicillin, 100 μg·mL^−1^ streptomycin (Sigma‐Aldrich) and fetal bovine serum (FBS; Japan Bio Serum, Hiroshima, Japan) at a concentration of 5%. The following supplements were also added to the medium: 2 nm triiodothyronine (Wako Pure Chemicals, Osaka, Japan), 10 ng·mL^−1^ recombinant human epidermal growth factor (Protein Express, Chiba, Japan), 5 μg·mL^−1^ transferrin (Gibco, Thermo Fisher, Waltham, MA, USA), 5 mg·mL^−1^ insulin (Gibco, Thermo Fisher), 0.4 mg·mL^−1^ hydrocortisone (Wako), 1 nm cholera toxin (Calbiochem, Darmstadt, Land Hessen, Germany) 9.

Neonatal NHEK (Lonza, Basel, Switzerland) were cultured on polystyrene nonpyrogenic cell culture dishes (Corning, One Riverfront Plaza, NY, USA) using KGM‐GOLD medium (Lonza). Cell subcultures were passaged no more than twice. Subconfluent NHEK cells were removed from the cell culture dish using 1.25% trypsin‐EDTA (Sigma‐Aldrich). The harvested cells were seeded onto temperature‐responsive cell culture inserts (UpCell Insert: CellSeed, Tokyo, Japan, circle 23.1 mm in diameter) at a density of 3.0–4.0 × 10^4^ cells·cm^−2^ and cultured in KCM at 37 °C in the presence of 5% CO_2_. After 9–10 days, cultured cells were harvested as a NHEK cell sheet by lowering the temperature to 20 °C for 30 min.

Normal human dermal fibroblasts (NHDF) cells (Lonza) were embedded in collagen gels at a concentration of 1.0 × 10^5^ viable cells·mL^−1^ 10.

### Cell sheet adhesion assay

**Figure 1:**
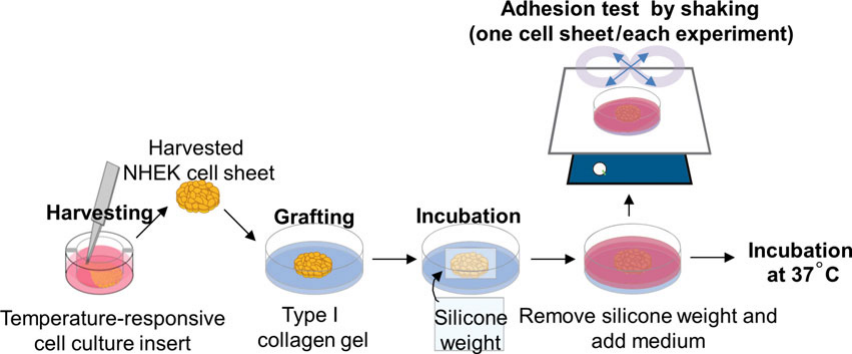
Schematic illustrations of the experimental method used. NHEK cell sheets harvested from the temperature‐responsive cell culture insert were grafted and cultured on a type I collagen gel. The cell sheet was adhered to the gel surface at 37 °C with a silicone weight. Cell sheet adhesion to the collagen gel surface was evaluated by stability to shaking and histology.

Normal human epidermal keratinocyte cell sheets were grafted prepared on type I collagen gels (Nitta Gelatin, Osaka, Japan). A silicone sheet (30 × 30 × 1 mm; ~ 0.6 g) covered with nylon mesh (Sansyo, Tokyo, Japan) was used to weigh down the cell sheet and facilitate cell adhesion to the hydrogels. After incubation at 37 °C for 5–60 min, the silicone sheet was removed and 2 mL of KCM was added to the culture dish. To ensure sufficient adhesion of cell sheets, samples were vigorously shaken in a 20‐mm‐wide figure‐eight motion at 100 r.p.m. using a shaker (Shake‐XR; Taitec, Tokyo, Japan) for 30 s. The adherent cell sheets were cultured for a further 3 weeks, and the behaviors of the epithelial cell sheet was examined using a time‐lapse phase‐contrast microscope equipped with a cell tracking system (Eclipse Ti, Nikon, Tokyo, Japan). Medium was replaced every 2 days. A schematic of these procedures is shown in Fig. 1.

### Particle image velocimetry (PIV)

To visualize cell migration patterns, we used particle image velocimetry (PIV) to measure the direction of each area between two time‐lapse phase‐contrast microscope images using the imagej software (National Institutes of Health, Bethesda, MD, USA) and the PIV plug‐in (The MathWorks, Natick, MT, USA) 11. The PIV plug‐in tracks pixels from microscopic images of migrating cells and enables quantitative visualization of direction and velocity.

### Immunohistochemistry (IHC)

Cell sheets were fixed at 4 °C using 4% paraformaldehyde (Muto Pure Chemicals, Tokyo, Japan) containing protease/phosphatase inhibitors (Cell Signaling, Danvers, MA, USA). The fixation time was for a minimum of 8 h or for a maximum of 2 weeks. The fixed samples were then embedded in paraffin. As controls, normal human oral epithelial tissues embedded in paraffin blocks were obtained from Analytical Biological Services (Wilmington, DE, USA). A human esophagus sample embedded in a paraffin block was provided by Nagasaki University with permission of the relevant Institutional Review Board. Paraffin embedded samples were cut into 5‐μm‐thick sections and dried overnight at 40 °C. Paraffin was removed from the sections with xylene and ethanol, and then washed with pure water. The sections were stained with hematoxylin and eosin (HE) according to the manufacturer's protocol (Muto Pure Chemicals).

For immunohistochemistry (IHC), the deparaffinized sections were treated with proteinase K (Dako, Santa Clara, CA, USA) or heated with citrate (Dako). To block endogenous peroxidase activity and prevent nonspecific reactions, the sections were incubated with a peroxidase‐blocking solution (Dako) and a chemically synthesized blocking solution (Nacalai Tesque, Kyoto, Japan) at room temperature for 1 h. The sections were then treated with primary antibodies diluted in blocking buffer at 4 °C overnight (Table S1). The following day, sections were washed with three times with PBS for 5 min each time. Samples were then incubated with horseradish peroxidase‐conjugated secondary antibodies (Dako) at room temperature for 1 h. The sections were washed again and then reacted with 3,3′‐diaminobenzidine (Dako) for up to 10 min to allow visualization. Nuclei were costained with hematoxylin.

To quantify IHC results, we used IHC Profiler plug‐in for imagej software (SourceForge, Mountain View, CA, USA) 12. The IHC Profiler can automatically classify antibody staining intensity into four levels (High positive, HP; Positive, P; Low Positive, LP; Negative, N), and show each value in percentage. The expression levels of the target proteins were finally determined by the balance of these percentage values according to the unique algorithm of the software.

### Quantitative real‐time PCR (qRT‐PCR) analysis

Quantitative real‐time PCR (qRT‐PCR) was performed according to a method previously described 13. Total RNA was extracted from cultured whole NHEK cell sheets using the RNeasy plus mini kit (Qiagen, Hilden, Germany), according to the manufacturer's protocol. Preparation of cDNAs was performed using a First strand cDNA Synthesis kit (Origene, Rockville, MD, USA), according to the manufacturer's protocol. PCR master mix included DNA polymerase (Applied Biosystems, Foster City, CA, USA), prepared cDNAs, and commercially available primers and TaqMan probes were mixed according to the manufacturer's protocol and performed using StepOnePlus real‐time PCR system (Applied Biosystems). TaqMan probes are shown as follows: *GAPDH*, Hs99999905_m1; *VIM*, Hs00185584_m1; *TWIST*, Hs00361186_m1; *FN1*, Hs00365058_m1; *CDH1*, Hs00170423_m1; *CDH2*, Hs00983056_m1; *COL17A1*, Hs00990036_m1; *MKI67*, Hs01032443_m1; *TP63*, Hs00978339_m1; *FLG*, Hs00856927_g1 (Applied Biosystems). We performed statistical analysis on graphpad prism 6.0 software (GraphPad Software, La Jolla, CA, USA) using a *t*‐test, significance being set at *P* < 0.05.

### Measurement of proliferation

Cell proliferation was analyzed using Ki67 staining. Fifteen paraffin sections at each of the three stages of culture (before grafting, Day 1 postgrafting, Day 7 postgrafting) were stained with Ki67 using an IHC method. In total, 3030 Ki67‐positive cells were counted as follows: before grafting (2152 Ki67‐positive cells), 1 day after grafting (327 Ki67‐positive cells), and 7 days after grafting (339 Ki67‐positive cells). We performed statistical analysis on graphpad Prism 6.0 software using ANOVA with a Tukey–Kramer *post hoc* test, significance being set at *P* < 0.05.

## Results

### Immunohistochemistry of harvested NHEK cell sheets

**Figure 2:**
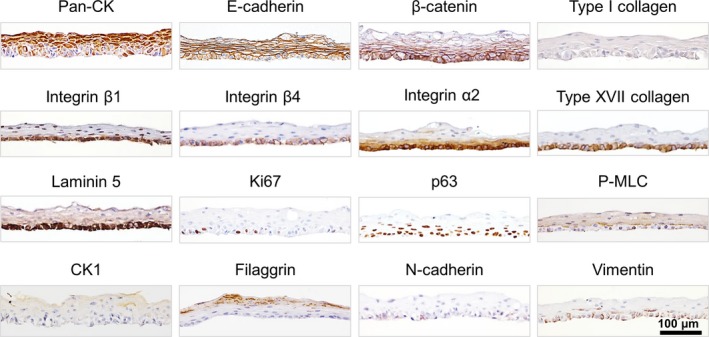
Immunohistochemical analysis of harvested NHEK cell sheets. The top of each panel is labeled with the protein of interest (see also Table S1).

Freshly detached NHEK cell sheets with a roughly circular shape were examined by IHC (Fig. 2). Since the tissue histology and target protein expression patterns are similar in human oral or esophageal tissues 14, these were used as positive controls in order to validate the antibodies (Fig. S1). To detect any EMT behavior, we used the epithelial markers: pan‐cytokeratin (Pan‐CK), E‐cadherin, and β‐catenin 15, 16, and the mesenchymal markers were also used: N‐cadherin and vimentin 17. Type I collagen was selected as a wound bed marker 18. Integrin β1, integrin β4, type XVII collagen (also known as BP180), and laminin 5 (also known as laminin 332) were used as markers of basal layer cells 19, 20. Cytokeratin 1 (CK1) and filaggrin were used as markers of the upper cell layer 21. To monitor cell proliferation, we examined nuclear proteins such as Ki67 and p63 22, 23. Phospho‐myosin light chain 2 (P‐MLC) was used to monitor cell dynamics such as cell migration and barrier function 24 (Table S1). NHEK cell sheets were harvested from the temperature‐responsive cell culture inserts 10 days after cultivation by reducing the temperature to 20 °C.

The harvested epithelial cell sheets were composed of several layers of epithelial cells all expressing cytokeratin (Fig. 2). E‐cadherin and β‐catenin were also present between cells in all layers of the cell sheet. P‐MLC was detected in the cytoplasm of the upper layer cells, suggesting a barrier function with actin.

On the other hand, the basal layer cells displayed distinctive features. These cells possessed a cuboidal shape, and expressed matrix receptor and adhesion proteins such as integrin β1, integrin β4, type XVII collagen, and laminin 5. On more detailed examination, laminin 5 and integrins were found to be not only at the basal side of cellular membrane but also at the cell–cell interfaces. Furthermore, a putative stem/progenitor marker p63 and proliferation marker Ki67 were expressed on the basal side of the cell sheet. In contrast, cells in the upper layer of the cell sheet expressed epidermal differentiation markers such as CK1 and filaggrin. These expression patterns were quantified by using IHC Profiler and summarized in Table S2.

### Characteristic change in the NHEK cell sheet after grafting

**Figure 3:**
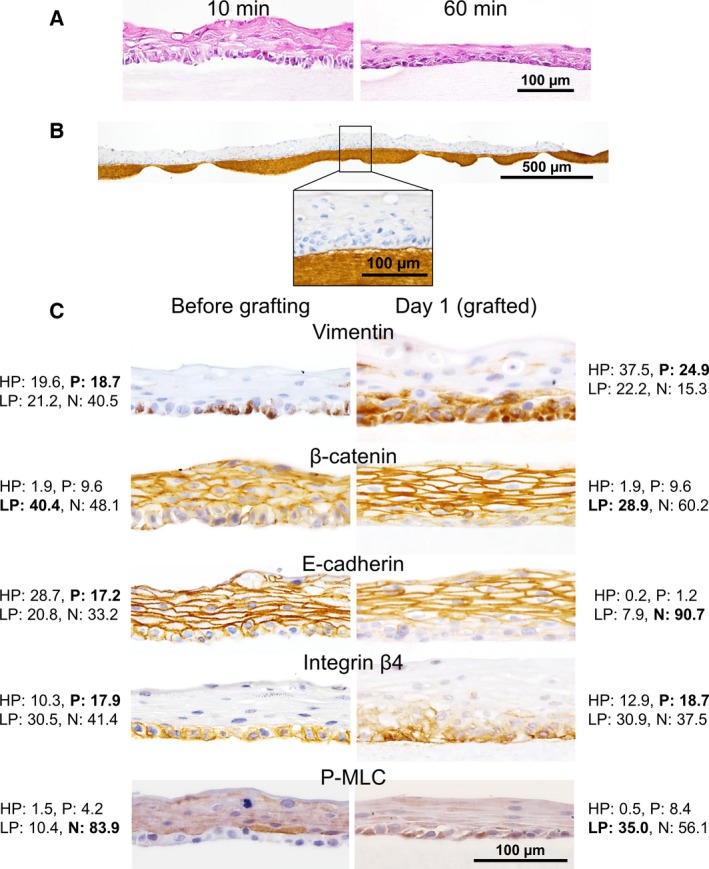
Histological evaluation of NHEK cell sheets on type I collagen gels. (A) Cell sheets weighed down on the collagen gel for 10 or 60 min were stained with HE. Gaps between the cell sheet and the collagen gel were notable in the sample weighed down for 10 min, whereas they were not present in the sheet weighed down for 60 min. (B) Anti‐type I collagen antibody staining was used to visualize the adhesion interface between the cell sheet and the gel. The cell sheet was weighed down for 60 min and then incubated for 1 day at 37 °C. (C) Immunohistochemical analyses of NHEK cell sheets before (left column) and 1 day after (right column) grafting. Collagen gels beneath the grafted cell sheets were not clearly visible (right column). Expression of vimentin, β‐catenin, E‐cadherin, Integrin β4, and P‐MLC is shown. The percentage values of HP, P, LP, and N are shown alongside each image. The final determination of the expression levels of the target proteins is presented in bold letters.

The adhesion of the cell sheet to the collagen gel was evaluated. Harvested cell sheets were gently placed on the surface of a hydrated collagen gel. In order to achieve firm contact between the cell sheet and the collagen gel, a silicone weight (~ 0.6 g) was placed on the cell sheet, and the entire construct was incubated at 37 °C on various culture‐time conditions (Fig. 1). Incubation at 37 °C for < 5 min led to unstable adhesion between the cell sheet and the collagen gel, resulting in cell sheet separation from the collagen gel during the paraffin embedding and sectioning procedures. However, more stable adhesion was accomplished with incubation times longer than 10 min (Fig. 3A). When the incubation was prolonged for 60 min, adhesion of the cell sheet and collagen gel could be clearly observed in a cross‐sectional image (Fig. 3A). Cell sheets incubated for more than 60 min were resistant to shear stress by vigorous shaking. Histological examination of the cell sheets grafted onto the collagen gel surfaces for 1 day, a time when cell migration was not evident (see the next section), also showed that the collagen gel and cell sheet were closely adhered (Fig. 3B). When the NHEK cell sheets was reversed and the apical surface was placed in contact with the collagen gel, stable adhesion was not observed, implying that the polarity of the cell sheet was established and that the initial adhesion of the cell sheet was likely due to the presence of ECM and matrix receptors.

The expression of several proteins was compared before and after grafting onto the collagen gel surface (Fig. 3C). Protein expression changes in the basal layer cells of the epithelial cell sheet were significantly different from the changes observed in the upper layer cells. The basal cells of the cell sheet positively expressed integrin β4 both before (HP, 10.2%; P, 17.8%; LP, 30.4%, N, 41.3%) and after grafting (HP, 12.9%; P, 18.7%; LP, 30.9%, N, 37.5%). Although the expression level did not change much, Integrin β4 was observed at cell–collagen surface as the matrix receptor particularly after grafting. E‐cadherin and β‐catenin expression was observed at cell–cell junctions in all upper layer cells both before and after the grafting. In contrast, the IHC Profiler analysis in basal layer of E‐cadherin was scored as negative (HP, 0.2%; P, 1.2%; LP, 7.9%, N, 90.7%) and β‐catenin was scored as LP (HP, 1.9%; P: 9.7%; LP, 40.3%; N, 48.1%). These results show that cell–cell junctions in each basal cell decreased. In addition, β‐catenin was not observed in nuclei of basal layer cells. Vimentin expression was positive in the basal layer both before (HP, 19.6%; P, 18.7%; LP, 21.2%, N, 40.5%) and 1 day after grafting (HP, 37.5%; P, 24.9%; LP, 22.2%, N, 15.3%). Moreover, although P‐MLC expression in basal layer was negative before grafting (HP, 1.4%; P, 4.2%; LP, 10.4%, N, 83.9%), it was LP 1 day after grafting (HP, 0.5%; P, 8.4%; LP, 35.0%, N, 56.1%). Overall, these results also suggest that basal layer cells acquired the ability to migrate along with EMT‐like events. These quantitation results of IHC in the basal layer before and 1 day after grafting are summarized in Table S3.

### Migration behavior of the NHEK cell sheet

The shape of the cell sheet and the movement of individual epithelial cells within it were recorded in the time‐course experiment. Immediately after detachment from the temperature‐responsive cell culture insert, the harvested cell sheets shrank isotropically to about 70% of the original size and a slight shrinkage persisted over the first 4 days on the collagen gel (Videos S1 and S2, Fig. S2). However, between the second and fourth days, the ‘inward’ shrinkage behavior of the cell sheet became ambiguous, and it synchronized with the ‘outward’ cell migration (Videos S2 and S3 see below). The size and circular shape of the entire cell sheet became similar to that observed immediately after detachment. Between Day 5 and 7, cell shrinkage could not be observed (Video S4).

**Figure 4:**
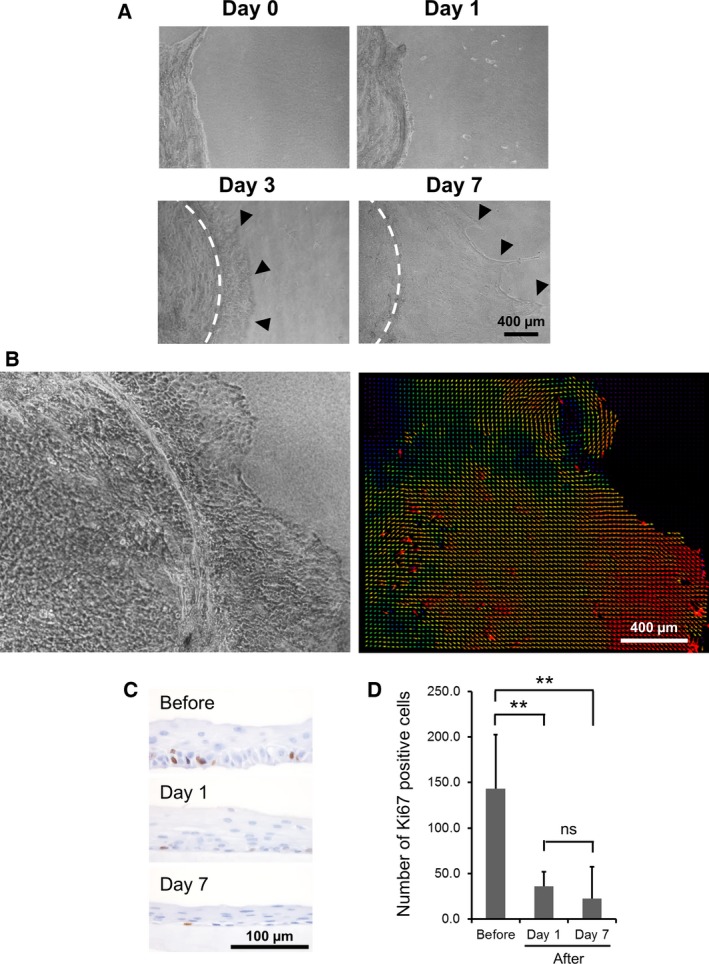
(A) Phase‐contrast microscopy images of epithelial cells migration from the periphery of the grafted cell sheet at Day 0, 1, 3, and 7 postgrafting. Arrowheads show the leading edges of the migrating cells. The white dotted lines show the periphery of the upper layer of the cell sheet. (B) Representative time‐lapse images taken 2–4 days postgrafting and PIV analysis of the direction of migration. Each colored arrows shows the cell velocity (red: 36.7 μm·h^−1^; orange: 30.0 μm·h^−1^; yellow: 20.0 μm·h^−1^; green: 13.3 μm·h^−1^; blue: 6.7 μm·h^−1^; purple: < 6.7 μm·h^−1^). (C) Immunohistochemical analysis of Ki67‐positive cells in cross‐sectional images of the cell sheets at three different time points (before, Day 1 and 7 postgrafting). (D) Number of Ki67‐positive cells in a cross‐section of the whole cell sheet at each time point. Mean ± SD (*n* = 5). **Statistical significance by ANOVA with Tukey–Kramer *post hoc* test; *P* < 0.01. ns, no significance.

Phase‐contrast microscopy revealed that cells migrated from the periphery of the cell sheet on Day 3 and 7 (Fig. 4A). Migrating cells were observed throughout almost the entire area of the cell sheet (Fig. S3). This phenomenon further supports the presence of EMT‐like events, which were suggested from the immunohistochemical analysis. Cells migrating from the periphery of the cell sheet moved forward and sometimes backward (see Videos S2 and S3). Precise observation using a PIV plug‐in showed that not all cells moved in the same direction. Rather, a number of cell‐clusters were found, and cells in clusters moved synchronously. However, PIV analysis shows that various directions of the movement were observed among different clusters. (Fig. 4B, Video S3). The presence of peripheral cells was not only caused by shrinkage of the upper layer but also by basal epithelial cell migration after EMT‐like events. No epithelial cells were observed in the collagen gel. It was apparent that the collective migratory movement of the basal cells was confined to the gel surface.

The average number of Ki67‐positive cells present in a given section of the cell sheet were 144 ± 74, 36 ± 29, and 23 ± 33 (mean ± SD, *n* = 5) before grafting, at Day 1, and at Day 7 after grafting, respectively (Fig. 4C,D). Cell proliferation did not occur along with cell migration because the Ki67‐positive cell frequency after grafting was significantly lower than before grafting (*P* < 0.01).

### Lateral distribution of marker proteins in the cell sheet after migration

**Figure 5:**
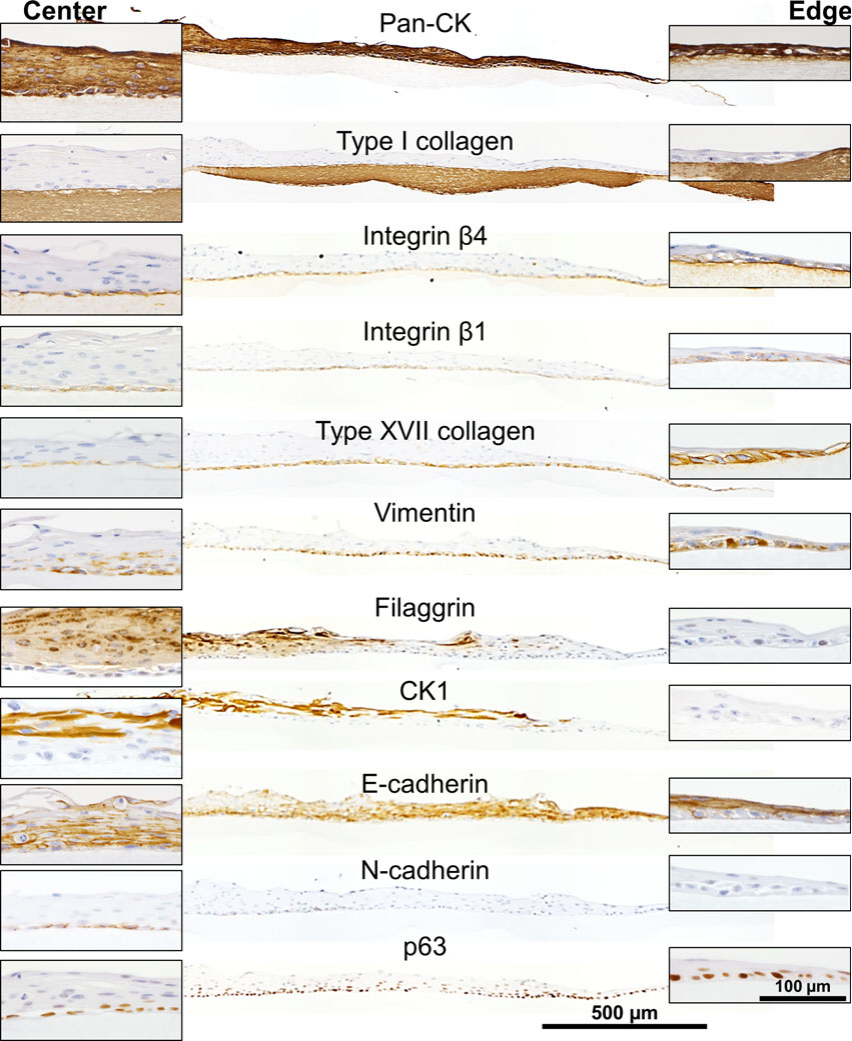
IHC of NHEK cell sheets on the type I collagen gels at Day 7 postgrafting. The center (left insets) and migratory leading edge (right insets) of the cells sheet are shown magnified. The top of each panel is labeled with the protein of interest.

Cell sheets grafted onto collagen gels and incubated for 7 days, a time by which cell migration was evident (see above), were examined for expression of numerous cell markers using IHC. The migratory leading edge of the cell sheet showed significant expression of basal cell markers namely integrin β1 and β4, type XVII collagen, and vimentin, whereas it did not express upper layer markers such as CK1 and filaggrin (Fig. 5), indicating that basal cells direct migration of the cell sheet. The expression of both type XVII collagen and vimentin was relatively low at the center of cell sheet. E‐cadherin was expressed at very low levels in basal cells, but was expressed at higher levels in the supra‐basal layer through the upper layer at the migratory leading edge. With the exception of filaggrin, the protein expression patterns were maintained over a period of 3 weeks (Fig. S4). These characteristic expression patterns within the cell sheet layers are indicative of EMT‐like events coupled with the result that the cell sheet kept a migratory motion (see above). Furthermore, intercellular spaces, which are associated with the dissolution of cell junctions and the acquisition of migratory function 25, were remarkable within the basal layer (Fig. S4). Moderate N‐cadherin expression was also detected in basal cells at the center of the cell sheet, also supporting the presence of EMT‐like events (Fig. 5). p63 was present in the nuclei of basal epithelial cells throughout the cell sheet. No cells were found to have migrated into the collagen gel.

### Gene expression analysis

Gene expression of the mesenchymal cell markers, vimentin (*VIM*), Twist (*TWIST1*), fibronectin (*FN1*), and N‐cadherin (*CDH2*), were analyzed by qRT‐PCR analysis. In addition, the gene expression of E‐cadherin (*CDH1*), type XVII collagen (*COL17A1*), Ki67 (*MKI67*), p63 (*TP63*), and filaggrin (*FLG*) were also analyzed to further substantiate the immunohistochemical data. For all genes examined, there was no significant difference in gene expression after grafting of the cell sheet compared to before grafting (Fig. S5, *P* > 0.05).

### Effect of feeder cell on the stratified squamous epithelial cell sheet

**Figure 6:**
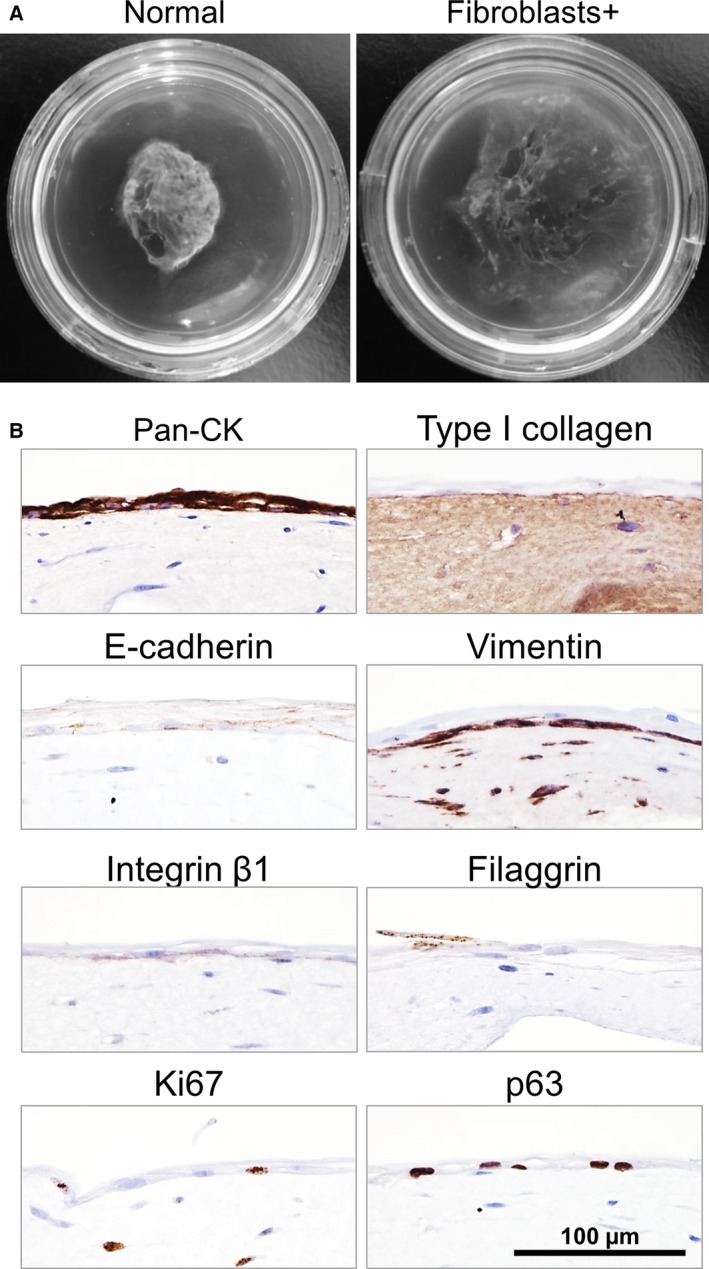
Impact of embedding NHDF cells in the collagen gel on the epithelial cell sheet. (A) Left hand panel: Epithelial cell sheet grafted without NHDF cells at 7 days postgrafting. Right hand panel: Epithelial cell sheet grafted in the presence of NHDF cells embedded in the collagen gel at 7 days postgrafting. (B) Immunohistochemical analyses of the NHEK cell sheets cultured on a collagen gel containing NHDF cells at 7 days postgrafting. The top of each panel is labeled with the protein of interest.

Normal human dermal fibroblasts were embedded in the collagen gel as feeder cells in order to imitate connective tissue structure because *in vivo* wound beds contain many fibroblasts. Although the shrinkage behavior of the cell sheet was also observed at first 2 days (Video S5), the epithelial cell sheet cultured thereon increased its migration activity and its diameter increased approximately two‐fold compared to a cell sheet cultured without NHDF (Fig. 6A, Video S6). As a result of this increase in cell sheet area, the cell sheet became noticeably thinner compared to cell sheets grafted on a non‐NHDF gel and had a transparent appearance 7 days after grafting. Although the thickness of the cell sheet changed, expression of marker proteins and the migration behavior of the cell sheet were not noted to be different between sheets cultured with and without NHDF feeder cells (Fig. 6B). Furthermore, difference in expression of the proliferation marker was also not observed.

## Discussion

Regenerative therapy using cellular constructs, in particular cell sheets, is a promising approach and will prevail as a standard for grafting therapy in the near future. In order to ensure safety and efficacy, precise studies on the dynamic behavior of the cellular grafts after grafting is essential. In this study, we designed an *in vitro* grafting model, which proved useful to study the time course of cellular events occurring in epithelial cell sheets after grafting.

Normal human epidermal keratinocyte cells are suitable as a model system, because the cell culture conditions have been already established and epithelial tissue‐like stratified squamous structures can be produced *in vitro* 7, 26. In this study, as expected, the NHEK cells formed polarized multiple layered cell sheets. The expression patterns of marker proteins were found to be different between the different layers within the sheet (Fig. 2). In the upper layers, Pan‐CK, E‐cadherin, CK1, and filaggrin were expressed, indicating that the NHEK cell sheet maintains its epithelial characteristics 27. Accordingly, the NHEK cell sheet shrank spontaneously after harvesting, pointing to the presence of strong cell–cell interactions that would be required for the barrier function of the upper layer. P‐MLC, which is known to be important in barrier function through its interaction with cadherin 28, 29, was also present in the upper layers of the cell sheet. Thus, we think that P‐MLC guides and orchestrates this shrinkage. Similarly, an epidermal sheet harvested from a cell culture dish using a proteolytic enzyme also exhibited shrinkage 9.

Laminin 5 is known to be secreted as an ECM protein by epithelial cells 30, 31, and it was found to be expressed at significant levels throughout the basal layer cells within the harvested cell sheet (Fig. 2) 5. Integrin β4, a protein with a hemidesmosome structure that functions as a receptor for ECM proteins, was also known for leading to cell migration in the wound‐healing process 20, 32, 33. Since the participation of integrins in cell–cell junctions has been reported 34, 35, laminin 5 might be recognized by integrin β4 not only at the basal side but also at the cell–cell interface before grafting (Fig. 2). In addition, integrin β4 located at the cell–collagen gel interface (Figs 3C and 5) could serve as a matrix receptor and transmit adhesion signals to basal cells. Consistently, adhesion via integrins was accompanied by P‐MLC expression in basal layer cells within the grafted cell sheet (Fig. 3C), because P‐MLC and actin stress fiber form a complex actomyosin structure to generate a traction force sufficient for cell motility 24, 36.

Before cell sheet fabrication, NHEK cells were subcultured using a low calcium medium to prevent differentiation. Under low calcium conditions, NHEK cells exhibit a mesenchymal‐like phenotype characterized by vimentin expression and intercellular junctions with low E‐cadherin expression at the cell–cell junction 13. Although the cell sheets were cultured in KCM containing a high concentration of calcium, they still exhibited the mesenchymal characteristic that vimentin was retained in the basal layer cells (Fig. 2). It has been reported that vimentin correlates with disruption of cell–cell interactions and the induction of epithelial cell migration 37. Therefore, the fact that vimentin remained in the basal layer cells postgrafting may have contributed to facilitating cell migration along with EMT 38, 39. The concept of EMT was first proposed by Hay in the 1980s. EMT, typically observed in embryogenesis (Type I), wound healing (Type II), and tumor development (Type III), was initially recognized as a phenomenon in which an epithelial cell lose its original characteristics and gains mesenchymal ones 17, 39, 40. Currently, the definition of EMT has been expanded and it is now regarded as a multistep processes ranging from the initial stage―epithelial cell―to the final stage―mesenchymal cell. Between these two stages, a stage with intermediate characteristics is referred to as partial EMT (also known as hybrid epithelial/mesenchymal or intermediate mesenchymal) 41, 42. It has also been revealed that EMT proceeds through a variety of pathways 39. Nuclear translocation of β‐catenin is a characteristic process mediated by the Wnt/β‐catenin signaling pathway. However, under some circumstances, other pathways may be involved. In partial EMT, for example, it has been shown that β‐catenin undergoes degradation after dissociation of the complex with E‐cadherin in the cytosol 39, 43; E‐cadherin also undergoes degradation.

From our data, β‐catenin was not observed in cellular nuclei (Figs 2 and 3C). Additionally, no significant change in mRNA expression of the various EMT markers occurred in the cell sheet after grafting. However, in addition to the acquisition of migration ability, the loss of E‐cadherin by its degradation and the expression of vimentin in the basal layer of the cell sheet strongly imply that partial EMT occurred after grafting, even though the extent of the transition and the pathway used remains unidentified.

The above‐mentioned cellular events showing evidence for partial EMT were maintained for over 3 weeks postgrafting (Fig. S4), which meets the time required for tissue regeneration via re‐epithelialization by cell sheet grafting, for example, in the esophagus 2. Several mechanisms have been proposed to describe the collective epithelial cell movement seen in spontaneous wound healing by re‐epithelialization. In the leap‐frog mechanism, supra‐basal cells overtake the basal layer cells 44. In the differentiation mechanism, cells individually take on new forms 45. A mechanism, whereby migration of basal cells occurred only at the edge of wound site, has also been proposed 46. In the tongue extension mechanism, basal layer cells lead comigration with the supra‐basal layer cells 47. From our data, at the leading edge of the stratified squamous epithelial cell sheet, supra‐basal migratory leading cells expressed integrin β1, β4, type XVII collagen, and vimentin, and following cells had some supra‐basal layers which expressed E‐cadherin (Fig. 5). Therefore, the migration of cells within the cell sheet following grafting appears to proceed through the tongue extension mechanism.

In conventional wound‐healing assays (e.g., scratch assay), a part of the confluent epithelial cells is removed, and re‐epithelialization of the defective area occurs. In these assays, individual epithelial cells show a directional migration toward the defective area 44, 45, 46, 47, 48. In the cell sheet grafting model, collective migration of the entire cell sheet moving back and forth was also observed (see Videos S2 and S3). Furthermore, it appeared that both the upper layer and the basal layer showed different movements. After 5 days culture on the collagen gel, shrinkage behavior was barely noticeable, probably because the upper layer cells started to peel off due to turnover. In fact, the filaggrin‐positive upper layer cell was not observed after 3 weeks of culture (Fig. S4).

It is plausible that fibroblasts embedded in the collagen gel enhanced the migration activity of the cell sheet, since they are known to secrete many cytokines including IL‐6, IL‐8, and G‐CSF 49, although which of these factor(s) is effective remains unclear. When ~ 50% of the ESD esophageal wound bed was covered with grafted epithelial cell sheets, the wound was completely re‐epithelialized and stenosis was prevented 2. It can be assumed that the cell sheet expanded and covered the remaining 50% of the wound bed with the assistance of resident fibroblasts. Moreover, epithelial cell sheets cultured on a fibroblast‐containing collagen gel showed a similar translucent appearance to those grafted on the esophageal wound bed 2 (Fig. 6), where the extent of transparency was so great that tissues and blood vessels underneath the cell sheets was easily observed 2, 6. Translucent or transparent epithelial cell sheets are a particular advantage in cornea grafting to improve visual acuity as demonstrated in our clinical regenerative therapies hitherto reported 1, 50. Whether peeling off of the upper layer due to turnover contributes to the formation of translucent cell sheet remains unclear.

**Figure 7:**
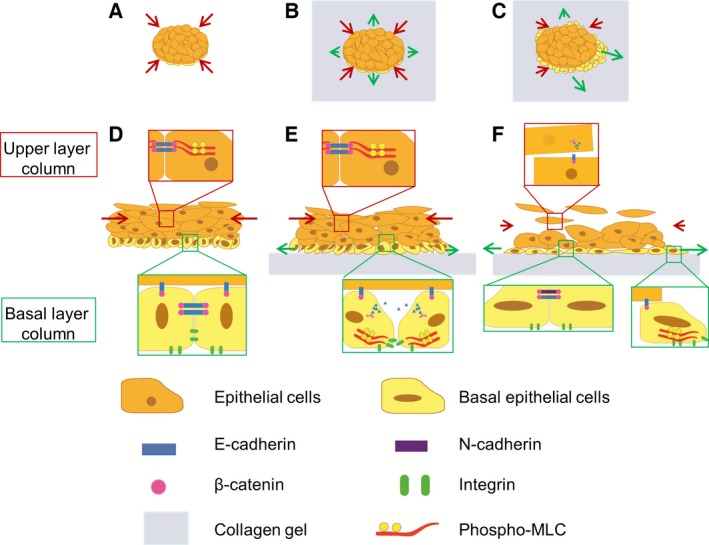
Schematic diagram of the time‐course transition of a stratified squamous epithelial cell sheet grafted onto the wound bed model. Three stages of the cell sheet behaviors are described; before grafting (A, D), the first 2 days after grafting (B, E), and the third and fourth days after grafting (cell‐migration stage) (C, F). Top‐down views of the three stages (A, B, C); cross‐sectional views of the three stages (D, E, F).

Based on the results of this study, the cellular events in cell sheet grafting are proposed to be classified into three stages along the wound‐healing process; harvesting of a cell sheet prior to grafting, a period immediately after grafting and for a further couple of days, and a period several days after grafting (Fig. 7). During the first stage, the harvested stratified squamous epithelial cell sheet before grafting has a distinct basal layer and upper layers. The basal layer retains matrix receptor and matrix proteins to allow adhesion on either a substrate or on wound beds, while the upper layers lead to shrinkage which can be ascribed to a large contractile force that reduce cell–cell gaps which leads to the formation of a barrier (Fig. 7A,D). During the second stage, over a period of couple of days postgrafting, cell–cell junctions are disrupted by the partial EMT in the basal layer cells (Fig. 7E), while the upper layer cells retain strong junctions, resulting in slight but continuous shrinkage of the cell sheet (Fig. 7B,E). In the third stage, due to the partial EMT, individual basal cells migrate and the basal layer cells lead migration of the cell sheet through a tongue extension mechanism; a similar process occurs in a spontaneous wound‐healing process by re‐epithelialization *in vivo* 51. The stratified structure of the epithelial cell sheet was well retained and was clearly observed when grafted onto a collagen gel, whereas the structure became hard to discern when the cell sheet was cultured onto a gel containing NHDF feeder cells presumably due to the remarkable extension of the cell sheet under these conditions. Thus, this simple model of grafting on a collagen gel enabled precise examination of the different behaviors of distinct layers within the squamous epithelial cell sheet.

This model will be also useful for further studies designed to improve the adhesion ability of cell sheets to wound beds, and to assess the humoral factors impacting the dynamic behavior of the cell sheet. This study contributes to our understanding of the cellular events occurring in tissue cultured for collective cellular events and regenerative therapy.

## Conclusion

In conclusion, a stratified squamous epithelial cell sheet can adhere to a hydrated collagen gel through basal layer proteins. After grafting, partial EMT occurs within the basal layer cells, and these basal layer cells can then migrate to expand like re‐epithelialization in wound healing process.

## Author contributions

YK designed and conducted experiments, analyzed data, and wrote the paper. NT and RT designed experiments, wrote the paper, and supervised this project. SK applied for permission from the institutional review board of Nagasaki University and provided human esophageal samples. MY supervised the work presented in this paper.

## Conflict of interest

Masayuki Yamato is a stakeholder of CellSeed Inc. Tokyo Women's Medical University receives research funding from CellSeed Inc.

## Supporting information


**Fig. S1.** Validation of antibodies using an immunohistochemical analysis of native human epithelial tissue.
**Fig. S2.** Shrinking behavior of the NHEK cell sheet after detachment.
**Fig. S3.** Migrating cells observed in the entire perimeter of the cell sheet at 7 days postgrafting.
**Fig. S4.** Immunohistochemical analyses of the NHEK cell sheets at 14 days (left column) and 21 days (right column) postgrafting.
**Fig. S5.** Quantitative RT‐PCR analysis of mRNA expression in the cell sheets pre‐ and postgrafting (mean ± SD, *n* = 3, *P* > 0.05).
**Table S1.** Antibodies used for immunohistochemistry.
**Table S2.** Quantitation of immunohistochemistry results of the harvested NHEK cell sheet.
**Table S3.** Quantitation of immunohistochemistry results in the basal layer of the NHEK cell sheet pre‐ and post grafting.

**Video S1.** Video image of the time‐lapse phase‐contrast microscopy analysis focusing on the cell sheet periphery was taken over a period of 2 days postgrafting.

**Video S2.** Video image of the time‐lapse phase‐contrast microscopy analysis focusing on the cell sheet periphery during 2–4 days postgrafting.

**Video S3.** Direction of cell migration in Video S2 analyzed using PIV.

**Video S4.** Video image of the time‐lapse phase‐contrast microscopy analysis showing the cell sheet migration during 5–7 days postgrafting.

**Video S5.** Video image of the time‐lapse phase‐contrast microscopy analysis of the cell sheet culture on a collagen gel containing NHDF cells over a period of 2 days postgrafting.

**Video S6.** Video image of the time‐lapse phase‐contrast microscopy analysis showing the cell sheet expansion on a collagen gel containing NHDF cells 2–4 days postgrafting.
