# Corrigendum to: Cellular events and behaviors after grafting of stratified squamous epithelial cell sheet onto a hydrated collagen gel

**DOI:** 10.1002/2211-5463.12688

**Published:** 2019-07-04

## Abstract

The original article [1] contained a typo in the ‘Construction of NHEK cell sheets’ section of the Materials and methods. The concentration of hydrocortisone was incorrectly written as 0.4 mg·mL ^−1^; the correct concentration of hydrocortisone is 0.4 μg·mL ^−1^.

**Reference**

1 Kasai Y, Takeda N, Kobayashi S, Takagi R and Yamato M (2017) Cellular events and behaviors after grafting of stratified squamous epithelial cell sheet onto a hydrated collagen gel. *FEBS Open Bio* **7**, 691–704. https://doi.org/10.1002/2211-5463.12213

The original article [1] contained a typo in the ‘Construction of NHEK cell sheets’ section of the Materials and methods. The concentration of hydrocortisone was incorrectly written as 0.4 mg·mL ^−1^; the correct concentration of hydrocortisone is 0.4 μg·mL ^−1^.

**Reference**

1 Kasai Y, Takeda N, Kobayashi S, Takagi R and Yamato M (2017) Cellular events and behaviors after grafting of stratified squamous epithelial cell sheet onto a hydrated collagen gel. *FEBS Open Bio* **7**, 691–704. https://doi.org/10.1002/2211-5463.12213

