# Supplementary material for: Cellular events and behaviors after grafting of stratified squamous epithelial cell sheet onto a hydrated collagen gel
Source: FEBS Open Bio. 2017 Mar 28;7(5):691–704. doi: 10.1002/2211-5463.12213 (PMC5407900; doi:10.1002/2211-5463.12213)
Supplement: Supplementary file 1 — Fig. S1. Validation of antibodies using an immunohistochemical analysis of native human epithelial tissue. Fig. S2. Shrinking behavior of the NHEK cell sheet after detachment. Fig. S3. Migrating cells observed in the entire perimeter of the cell sheet at 7 days postgrafting. Fig. S4. Immunohistochemical analyses of the NHEK cell sheets at 14 days (left column) and 21 days (right column) postgrafting. Fig. S5. Quantitative RT‐PCR analysis of mRNA expression in the cell sheets pre‐ and postgrafting (mean ± SD, n = 3, P > 0.05). Table S1. Antibodies used for immunohistochemistry. Table S2. Quantitation of immunohistochemistry results of the harvested NHEK cell sheet. Table S3. Quantitation of immunohistochemistry results in the basal layer of the NHEK cell sheet pre‐ and post grafting. [file FEB4-7-691-s001.pdf]

Y Kasai, et al.
Submitted to *FEBS Open Bio*

Supplemental informations

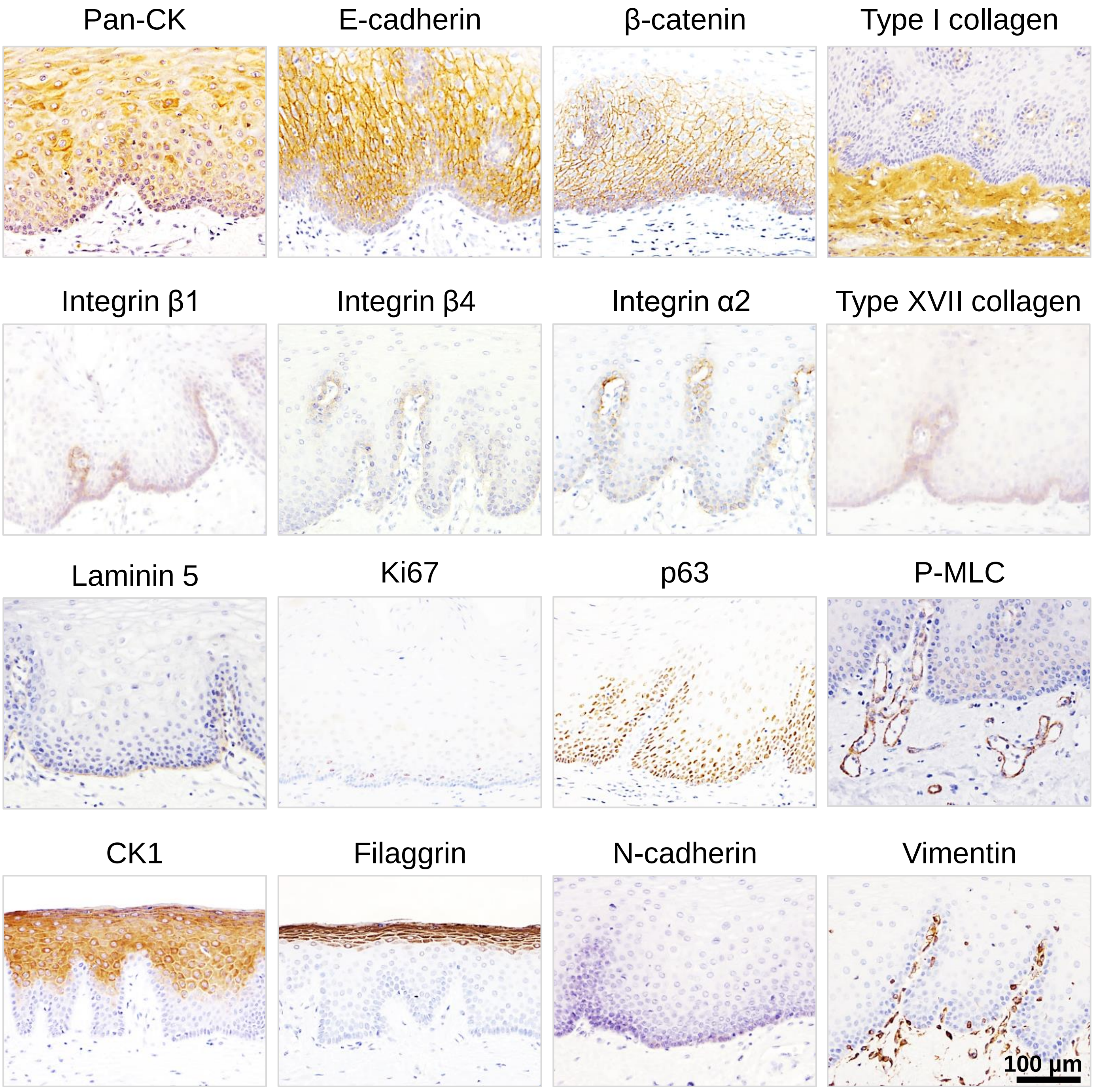

Fig. S1. Validation of antibodies using an immunohistochemical analysis of native human epithelial tissue. The top of each panel is labeled with the protein of interest (see also Table S1).

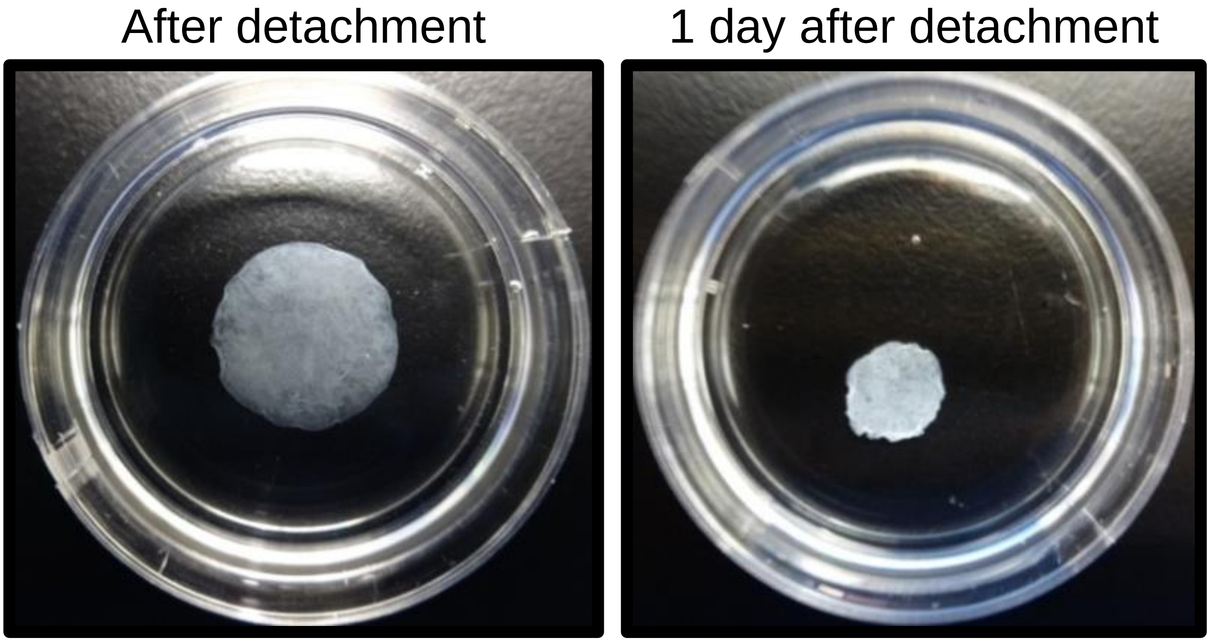

Fig. S2. Shrinking behavior of the NHEK cell sheet after detachment. Immediately after detachment from the temperature-responsive cell culture insert (left) and 1 day after detachment without grafting (right).

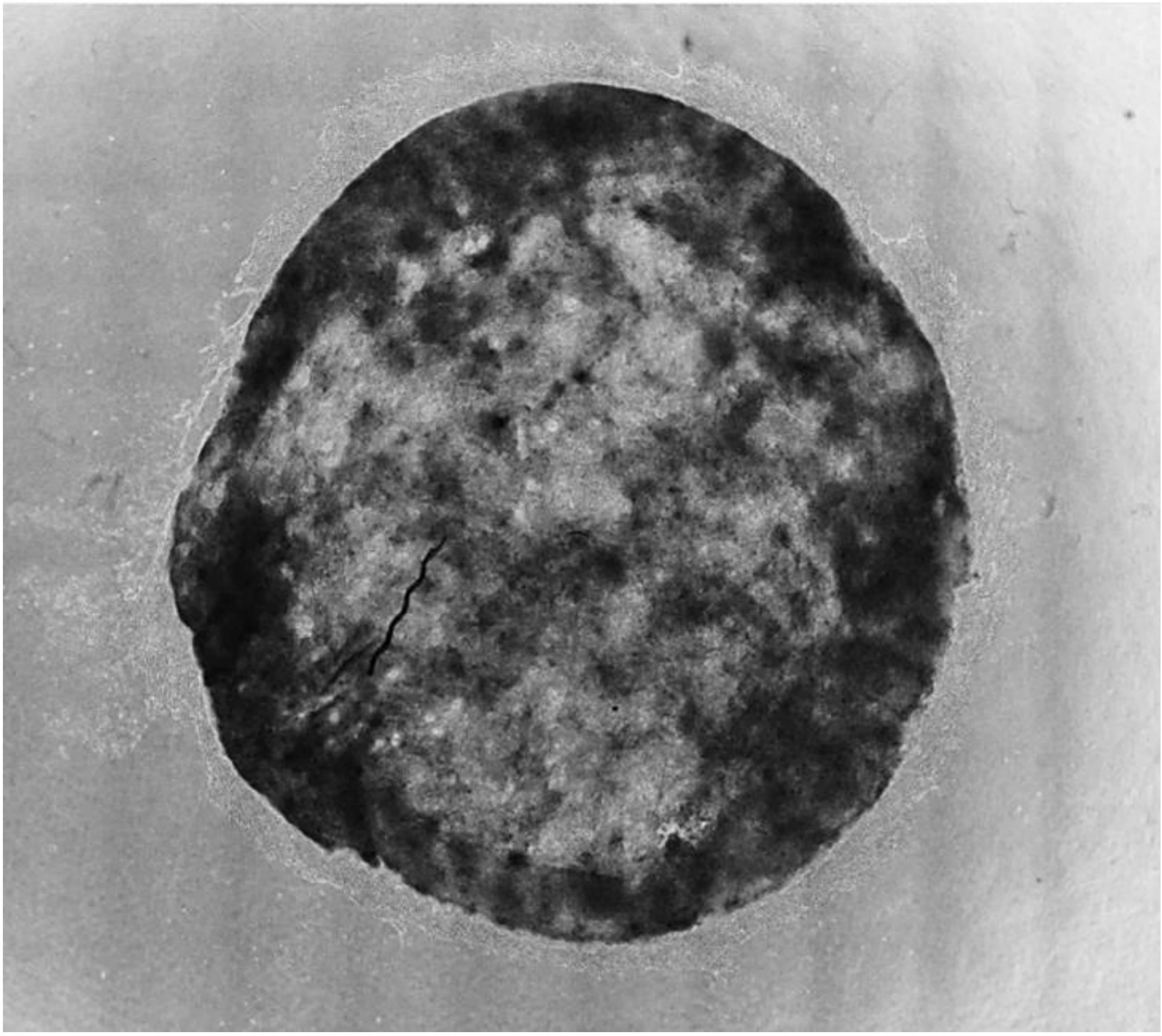

Fig. S3. Migrating cells observed in the entire perimeter of the cell sheet 7 days post-grafting.

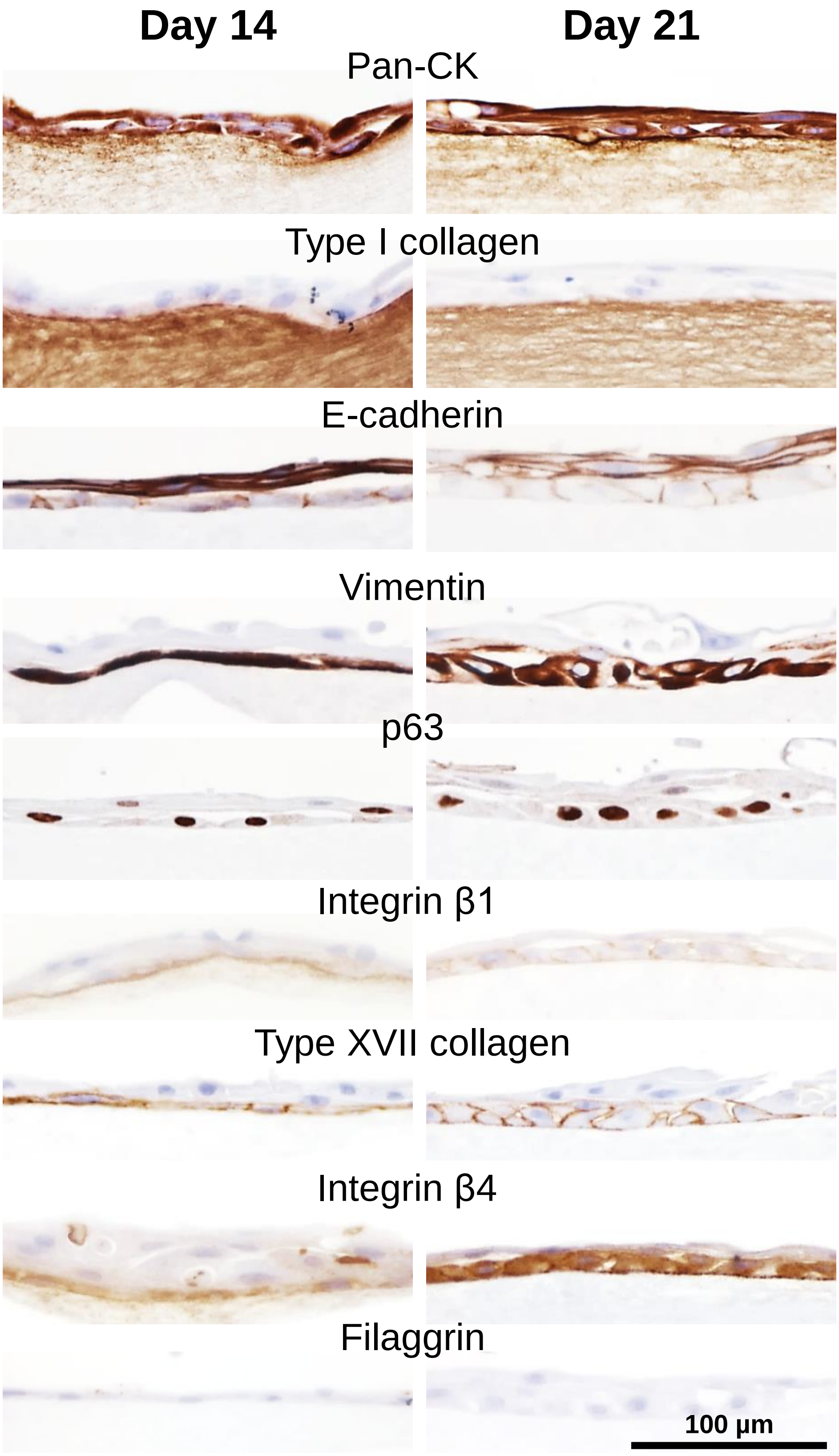

Fig. S4. Immunohistochemical analyses of the NHEK cell sheets at 14 days (left column) and 21 days (right column) post-grafting. The top of each panel is labeled with the protein of interest.

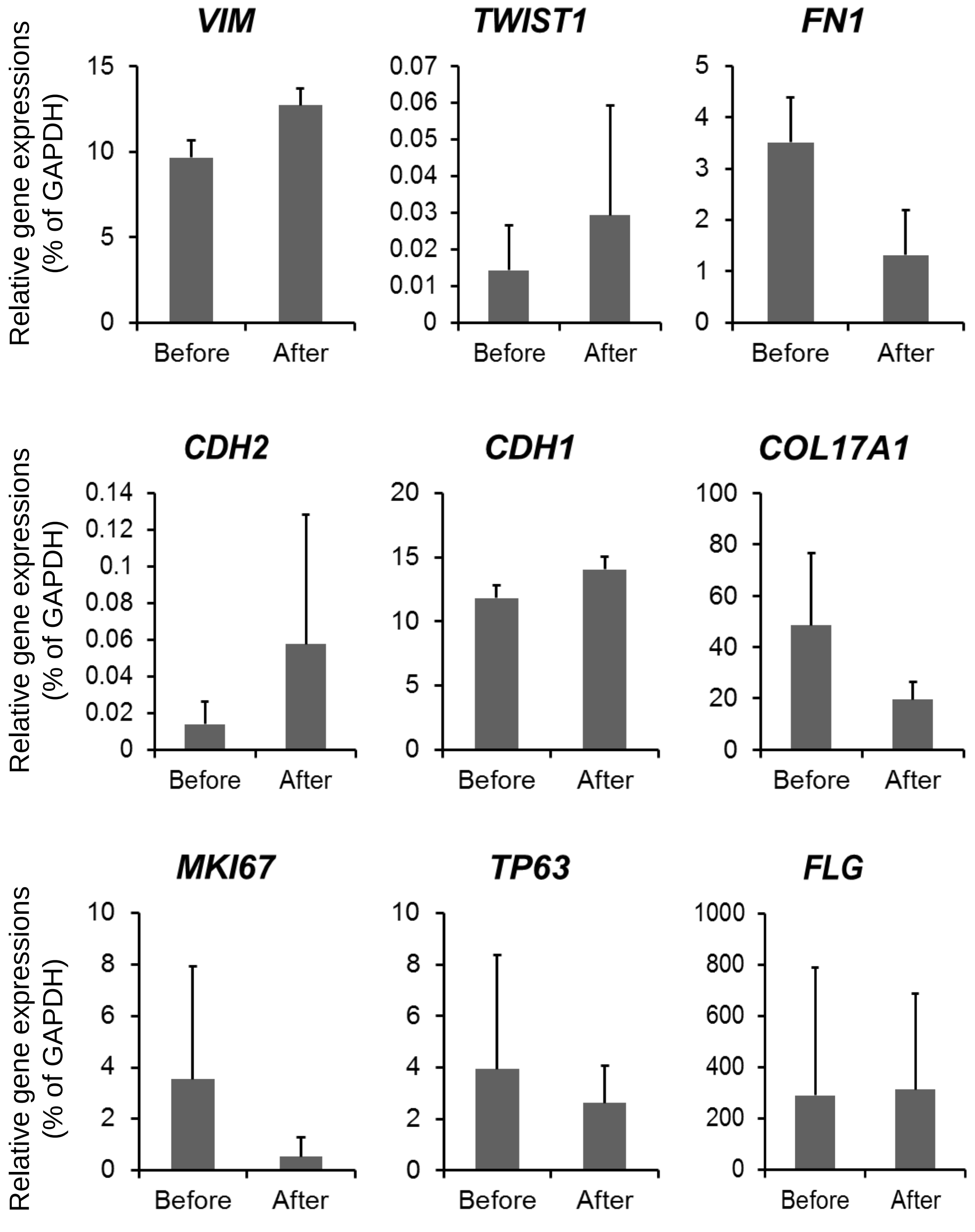

Fig. S5. Quantitative RT-PCR analysis of mRNA expression in the cell sheets pre- and post-grafting. The top of each panel is labeled with the mRNA of interest. The expression level of each mRNA was normalized using the levels GAPDH mRNA. Mean ± S.D. (n=3). Analysis of the gene expression data revealed no statistical difference in mRNA levels between pre- and post-grafting ($p > 0.05$).

Table S1. Antibodies used for immunohistochemistry.

Name	Clone	IHC dilution	Cat No./Vendor	Function	Reference
Pan-cytokeratin	AE1/AE3	1/100	ab27988, Abcam	Epithelial intermediate filament (Epithelial marker)	15
E-cadherin	NCH-38	1/100	M3612, Dako	Epithelial cell-cell adhesion marker	16
β-catenin	β-catenin	1/100	M3539, Dako	Epithelial cell-cell adhesion and transcription factor of Wnt/β-catenin pathway for EMT (Epithelial marker)	16, 46, 47
N-cadherin	D4R1H	1/100	13116, Cell signaling	Epithelial cell-cell adhesion protein (Mesenchymal marker)	17
Vimentin	V9	1/50	M0725, Dako	Fibroblast intermediate filament (Mesenchymal marker)	17, 32, 33
Type I collagen	-	1/400	ab34710, Abcam	Submucosal tissue (Wound bed marker in this study)	18
Integrin β1	-	1/100-200	4706, Cell signaling	Matrix protein receptor (Basal cell layer marker)	19
Integrin β4	-	1/200	4707, Cell signaling	Matrix protein (Laminin) receptor (Basal cell layer marker)	19, 20, 29
Integrin α2	EPR5788	1/400	ab133557, Abcam	Matrix protein receptor (Basal layer cell marker)	19
Integrin α5	-	1/400-500	4705, Cell signaling	Matrix protein receptor (Basal layer cell marker)	19
Type XVII collagen	NC16A-3	1/30	ab79878, Abcam	Matrix protein receptor (Basal layer cell marker)	19, 20
Laminin 5	-	1/100	ab14509, Abcam	Hemidesmosome structure and adhesion protein (Basal layer cell marker)	19, 20, 30
Cytokeratin 1	34βB4	1/100	NCL-CK1, Leica	Differentiated epithelial intermediate filament (Upper cell layer marker)	21
Filaggrin	FLG01	1/100	MA5-13440, Thermo fisher	Differentiated epithelial cell marker (Upper layer cell marker)	21
p63	4A4	1/50	sc-8431, Santa cruz	Transcription factor fof epithelial stem/progenitor cells(Cell activity marker)	23
Ki67	MIB-1	1/100	M7240, Dako	Cell proliferation factor (Cell proliferation marker)	22
Phospho-myosin light chain 2 (Thr 18/Ser 19)	-	1/50	3674, Cell signaling	Cell mortion protein with actin (Cell dynamics marker for migration and barrier function as actomyosin)	24, 28, 29

Table S2. Quantification of immunohistochemistry results of the harvested NHEK cell sheet.

	Upper				Basal			
	HP	P	LP	N	HP	P	LP	N
Pan-cytokeratin	**52.0**	17.3	17.4	13.3	37.6	**9.1**	15.6	37.3
E-cadherin	37.8	**19.0**	16.8	26.3	28.8	**17.1**	20.8	33.2
β-catenin	19.3	**28.4**	27.8	24.6	1.9	9.7	**40.4**	48.1
N-cadherin	0.0	0.0	2.2	**97.8**	0.0	0.5	8.8	**90.7**
Vimentin	1.3	7.1	22.1	**69.5**	19.6	**18.7**	21.2	40.5
Type I collagen	0.0	0.0	0.0	**100.0**	0.0	0.0	1.8	**98.2**
Integrin β1	1.0	3.9	8.6	**86.5**	18.2	**19.8**	14.1	47.9
Integrin β4	0.0	1.6	15.5	**82.8**	10.3	**17.9**	30.5	41.4
Integrin α2	0.6	5.1	16.8	**77.5**	**64.9**	22.0	8.3	4.9
Type XVII collagen	0.2	2.9	9.2	**87.7**	**63.5**	24.2	6.9	5.4
Laminin 5	0.0	1.2	24.4	**74.3**	40.7	**17.1**	28.4	13.8
CK1	0.1	0.3	3.4	**96.3**	0.0	0.0	1.6	**98.4**
Filaggrin	7.2	11.0	**23.1**	58.8	1.3	2.1	3.7	**93.0**
Ki67	0.0	0.0	0.0	**100.0**	19.6	**13.2**	19.9	47.2
p63	25.7	**13.0**	20.1	41.2	29.7	**19.1**	19.6	31.6
Phospho-myosin light chain 2 (Thr 18/Ser 19)	1.1	9.6	**57.1**	32.2	1.5	4.2	10.4	**83.9**

HP: Percentage contribution of High Positive, P: Percentage contribution of Positive, LP: Percentage contribution of Low Positive, N: Negative. The final determination of the expression levels of the target proteins are presented in bold letters.

Table S3. Quantification of immunohistochemistry results in the basal layer of the NHEK cell sheet before and after grafting.

	Before				Day 1			
	HP	P	LP	N	HP	P	LP	N
Integrin β4	10.3	**17.9**	30.5	41.4	12.9	**18.7**	30.9	37.5
E-cadherin	28.7	**17.2**	20.8	33.2	0.2	1.2	7.9	**90.7**
β-catenin	1.9	9.6	**40.4**	48.1	1.8	9.2	**28.9**	60.2
Vimentin	19.6	**18.7**	21.2	40.5	37.5	**24.9**	22.2	15.3
Phospho-myosin light chain 2 (Thr 18/Ser 19)	1.5	4.2	10.4	**83.9**	0.5	8.4	**35.0**	56.1

HP: Percentage contribution of High Positive, P: Percentage contribution of Positive, LP: Percentage contribution of Low Positive, N: Negative. The final determination of the expression levels of the target proteins are presented in bold letters.
